# The P2X7 Receptor, a Multifaceted Receptor in Alzheimer’s Disease

**DOI:** 10.3390/ijms241411747

**Published:** 2023-07-21

**Authors:** Kaitryn E. Ronning, Paul-Alexandre Déchelle-Marquet, Yueshen Che, Xavier Guillonneau, Florian Sennlaub, Cécile Delarasse

**Affiliations:** INSERM, CNRS, Institut de la Vision, Sorbonne University, F-75012 Paris, France; kaitryn.ronning@inserm.fr (K.E.R.); paul-alexandre.dechelle-marquet@inserm.fr (P.-A.D.-M.); yueshen.che@inserm.fr (Y.C.); xavier.guillonneau@inserm.fr (X.G.); florian.sennlaub@inserm.fr (F.S.)

**Keywords:** purinergic receptor, Alzheimer’s disease, inflammation, microglia, cytokines, chemokines

## Abstract

Alzheimer’s disease (AD) is a progressive neurodegenerative disease characterized by impaired episodic memory and two pathological lesions: amyloid plaques and neurofibrillary tangles. In AD, damaged neurons and the accumulation of amyloid β (Aβ) peptides cause a significant release of high amounts of extracellular ATP, which acts as a danger signal. The purinergic receptor P2X7 is the main sensor of high concentrations of ATP, and P2X7 has been shown to be upregulated in the brains of AD patients, contributing to the disease’s pathological processes. Further, there are many polymorphisms of the *P2X7* gene that impact the risk of developing AD. P2X7 can directly modulate Aβ plaques and Tau protein lesions as well as the inflammatory response by regulating NLRP3 inflammasome and the expression of several chemokines. The significant role of microglial P2X7 in AD has been well established, although other cell types may also be important in P2X7-mediated mechanisms. In this review, we will discuss the different P2X7-dependent pathways involved in the development of AD.

## 1. The Purinergic Receptor P2X7

The purinergic receptor P2X7 is an ATP-gated cation channel that allows the influx of Na^+^ and Ca^2+^ and efflux of K^+^ and the formation of a non-selective pore allowing the entry of molecules up to 900 Da. Unlike other P2 receptors, P2X7 has a lower sensitivity to ATP and thus requires high levels of extracellular ATP (>0.1 mM) for activation [1,2]. Normally, there is a delicate balance of ATP concentrations between the intracellular range (3–10 mM) and the extracellular range (20–50 nm). Therefore, the release of ATP at these high levels serves as a danger signal when it is released by damaged or stressed cells, and P2X7 then detects these dramatic increases in extracellular ATP [3]. The properties of P2X7 can depend on the presence of ATP and ATP levels [4]. In mouse neural progenitor cells, P2X7 is involved in phagocytosis, cell proliferation, or cell death with the increasing concentration of extracellular ATP. However, P2X7-deficient mice are healthy and display no overt neurological phenotypes, suggesting that P2X7 does not play a major role in healthy central nervous system function [5]. Instead, the functions of P2X7 appear to be most important during pathological processes in the presence of a high amount of ATP. In particular, P2X7 has garnered significant attention in the field of immunology due to its broad expression in immune cells and its main involvement in inflammatory processes [6]. It acts as a key component in the activation of the inflammasome NLRP3 (NOD-like receptor family, pyrin domain containing 3), leading to the activation of caspase 1 and the release of pro-inflammatory cytokines interleukin-1β (IL-1β) and IL-18 [7,8]. Stimulating P2X7 also triggers the release of various pro-inflammatory substances like TNFα [9], IL-6 [10], CCL2 [11], and an excitotoxic level of glutamate [12,13] and the production of reactive oxygen species (ROS) [14,15]. These mediators contribute to neuroinflammation, reactive gliosis, and cell death. Prolonged P2X7 activation can also lead to cell death by apoptosis or lysis/necrosis depending on the cell type expressing it [8]. Interestingly, P2X7 in monocytes and microglia exhibits scavenger activity in the absence of ATP and serum [16]. The various functions associated with the properties of P2X7 have been shown to be implicated in a wide range of neurological and neurodegenerative disorders including Alzheimer’s disease (AD), suggesting that this receptor may be involved in pathological processes [17].

## 2. Alzheimer’s Disease

AD is a neurodegenerative disease associated with age-related cognitive deficits, especially memory loss. AD is characterized by the presence in the brain of amyloid plaques and neurofibrillary lesions, leading to synaptic deficits. Amyloid plaques are formed by the accumulation of extracellular aggregates of β-amyloid (Aβ) peptides, resulting from the sequential proteolysis of the amyloid precursor protein (APP) by β and γ secretases. In contrast to Aβ peptides, the soluble fragment of APP (sAPPα), generated by α-secretase, appears to have neurotrophic and neuroprotective properties [18]. Neurofibrillary lesions consist of intraneuronal fibrillar aggregates of hyperphosphorylated and abnormally phosphorylated Tau proteins. Tau, a microtubule-associated protein, plays a critical role in stabilizing the microtubule network and facilitating the transport of substances along axons. The progression of the tau pathology in the brain, according to Braak stages, correlates with cognitive impairments in AD patients [19], suggesting an instrumental role in underlying synaptic dysfunctions.

## 3. The P2X7 Purinergic Receptor and AD

P2X7 levels are elevated in AD, and it has been strongly implicated in AD, particularly due to its involvement in inflammatory processes.

### 3.1. P2X7 Expression

The initial indication that P2X7 might play a role in AD came from its elevated expression in both animal models of AD and AD patients. Increased levels of *P2rx7* mRNA and P2X7 protein were first demonstrated in different mouse models of AD, specifically the transgenic mouse lines that develop amyloid plaques Tg2576 (Prnp-HuAPP*KM670/671NL) [14], APPSwe/PSEN1dE9 (Prnp-Mo/HuAPP*KM670/671NL, Prnp-HuPSEN1*ΔE9) [15], APPPS1 (Thy1-HuAPP*KM670/671NL, Thy1-HuPSEN1*L166P) [20], and J20 (PDGF-HuAPP*KM670/671NL and V717F) [21]. Additionally, the expression of P2X7 significantly rises in the rat’s brain after intra-hippocampal injection of Aβ peptides, indicating that Aβ peptides themselves can induce the upregulation of P2X7 [22]. Given the importance of Tau tangles in the pathophysiology of AD, the interaction between P2X7 and these lesions has also been investigated. In the mouse models P301S (Prnp-HuMAPT*P301S, also known as PS19) and THY-Tau22 (Thy1-HuMAPT*G272V and P301S), increased levels of P2X7 were specifically associated with Tau pathology [23,24]. In further support of an increase in the P2X7 protein in models of AD, an iodinated radiotracer specific to P2X7 ([123I]TZ6019) has been developed, which revealed a 35% higher binding in the brain of P301S mice compared to control mice [25]. Furthermore, a significant upregulation of P2X7 has been observed in the cortex and hippocampus of AD patients, both in the plaque cores and surrounding amyloid lesions, as well as in proximity to neurofibrillary degeneration [20,21,22,24]. Finally, increased P2X7 levels have also been identified in the brains of patients with other various tauopathies, including frontotemporal lobar degeneration, Pick’s disease, and progressive supranuclear palsy [23,24], further supporting the link between Tau and P2X7. Interestingly, while P2X7 is upregulated in response to AD-specific pathological lesions, the following activation of the receptor by pathological levels of extracellular ATP may in turn further contribute to the progression of AD.

### 3.2. P2X7 and Genetic Risk Factors

The familial form of AD occurs due to mutations in either the *APP* gene or genes such as *PSEN1* and *PSEN2*, which encode enzymes responsible for generating the Aβ peptide. Other genetic risk factors are also associated with AD, the most significant being the allelic form of APOE. In addition, genome-wide association studies (GWASs) have highlighted other genes implicated in inflammatory processes, suggesting a significant role of inflammation in neurodegenerative processes during AD [26].

The human *P2X7* gene exhibits over 150 non-synonymous single nucleotide polymorphisms (SNPs) [27]. These SNPs can impact a wide variety of P2X7 properties such as agonist-binding affinity [28], trafficking to the cell surface [29], ion channel activity [30], and permeability of the pore [6,31] (key AD-relevant SNPs summarized in Table 1).

Two of the most studied *P2X7* SNPs change the formation and function of the channel pore. One of these is the 1513A>C polymorphism, which causes the amino acid change Glu496Ala in the carboxyl-terminal tail of P2X7 [32]. While the channel properties remain unaffected, this polymorphism confers a loss of function, affecting pore formation and cell death induction [32,34]. Interestingly, this SNP has also been associated with other inflammatory pathologies such as chronic lymphocytic leukemia and tuberculosis [35,36]. Another is the SNP 489C>T, which causes the amino acid change His155Tyr in the extracellular loop of P2X7 and confers a gain of function, increasing Ca^2+^ influx and pore formation [33]. The frequency of both of these SNPs was analyzed in 84 AD patients and 148 age-matched healthy controls. The presence of the 1513A>C allele (Glu496Ala) (loss of function) decreased the risk of developing AD by approximately four times in the absence of the 489C>T polymorphism [37], suggesting that some specific property of P2X7, potentially pro-inflammatory, may contribute to the development of AD.

P2X7 has a scavenger activity in the absence of serum, and this can also be affected by genetic polymorphisms. Specifically, a P2X7 (Gly150Arg)—P2X4 (Tyr315Cys) haplotype is associated with loss of P2X7-mediated phagocytosis [38]. This Gly150Arg mutation is also localized in the extracellular loop of P2X7. These findings indicate that impaired phagocytosis, which contributes to the defect in Aβ peptide clearance and leads to the increased accumulation of aggregates in the brain of AD patients, may in part be caused by P2X7 dysfunction [16].

In contrast to the above, a recent study determining a polygenic risk score based on 12 *P2X7* SNPs and 1 *P2X4* SNP in a cohort of 902 subject controls and 328 AD patients did not provide evidence of an association between *P2X7* and the risk of developing AD [39]. However, identifying specific single nucleotide polymorphisms (SNPs) is not easy due to the large number of *P2X7* variants that can be associated with gain or loss of functions. Furthermore, some of these variants are very rare in the general population, requiring a large number of subjects to be studied in order to demonstrate their potential involvement. In another recent study, an analysis was performed specifically on the frequency of the loss-of-function 1513A>C and 1405A>G SNPs and the gain-of-function 489C>T and 1068G>A SNPs in the aged population [40]. The authors highlighted a significant increase in the hypomorphic 1513CC SNP frequency with age in a European/USA Caucasian cohort. However, no correlation was observed when subjects from more countries were included in the analysis. These findings suggest that in low-income countries, having a functional P2X7 might be beneficial to protect against infections such as Plasmodium, Mycobacterium, and Chlamydia, while in high-income countries with an older population, an anti-inflammatory P2X7 phenotype is selected for, conferring protection against chronic inflammatory diseases such as AD [40].

Altogether, these genetic analyses suggest that P2X7 may play a role in the development of AD, particularly through its involvement in the immune system. However, these studies have not been able to determine the exact role of P2X7 in these inflammatory responses during the course of the disease.

### 3.3. P2X7 and Amyloid Plaques

One of the characteristic lesions found in AD, amyloid plaques, is specifically composed of Aβ peptides. Aβ peptides derive from the amyloidogenic processing of APP by β and γ secretases, while alternative cleavage by α secretase produces a soluble neurotrophic and neuroprotective fragment, sAPPα, which prevents the formation of Aβ peptides [18]. P2X7 can affect Aβ build-up via this and other pathways. In our in vitro studies, we demonstrated that short-term stimulation (less than 30 min) of P2X7 activates an α-secretase enzyme, which cleaves APP at a specific site within the Aβ peptide sequence [41]. This P2X7-dependent non-amyloidogenic processing involved the activation of the Rho kinase, MAP kinases ERK1/2, and JNK, leading to the recruitment of Ezrin, Radixin, and Moesin proteins and subsequent PI3 kinase activity [41,42]. Conversely, Leon-Otegui et al. showed in their in vitro study that prolonged activation of P2X7 (4 h) has the opposite effect, resulting in a decrease in α-secretase activity through GSK3 kinase and an increase in the production of Aβ peptides [43,44].

In vivo in J20 mice, an amyloid mouse model, the administration of the P2X7 antagonist brilliant blue-G (BBG) for 4 months resulted in a reduction in Aβ load, which is correlated with GSK3 activity as observed in vitro [44]. The effects of knocking out P2X7 in another AD mouse model, APPPS1 mice, also led to a decrease in Aβ plaques and Aβ peptide levels, and this effect was unlikely to be attributed to the modulation of APP cleavage [20].

The inconsistencies sometimes observed between results obtained in vitro and in vivo could be explained by the diverse roles of P2X7 in the brain, which depend on the local ATP concentration as well as cofactors and the activation state of P2X7-expressing cells [8]. Despite some differences, most studies suggest that inhibiting P2X7 in a pathological context decreases the accumulation of Aβ peptides.

### 3.4. P2X7 and Tauopathy

The impact of P2X7 invalidation on the development of Tau pathology has also been assessed. In THY-Tau22 mice, the absence of P2X7 had a mild effect on Tau phosphorylation in the hippocampus [23]. In another Tau mouse model, P301S Tau mice, treatment at 3 months or 9 months of age with the specific P2X7 antagonist GSK1482160 or knockout of P2X7 reduced the accumulation of misfolded Tau protein and the rate of intraneuronal Tau protein phosphorylation in the hippocampus [24,45]. In agreement with the hypothesis that P2X7 may promote Tau accumulation, increased levels of P2X7 in P301S mice exacerbated Tau pathology [24]. There are multiple pathways/mechanisms by which P2X7 may augment Tau pathology. In P301S mice, the involvement of P2X7 in Tau phosphorylation is related at least in part to P2X7-dependent GSK3 activity, similar to what has been observed in P2X7-dependent modulation of Aβ in vitro and in J20 mice [24]. An additional possible mechanism by which P2X7 promotes Tau accumulation is that the activation of P2X7 induces the release of exosomes containing Tau [45]. Taken together, these findings suggest that inhibiting P2X7 could have a beneficial impact on Tau lesions as well as the Aβ plaques.

### 3.5. P2X7 and Synaptic Functions

The presence of Aβ plaques and neurofibrillary tangles contributes to the impairment of synaptic functions in AD. Recent studies have raised the possibility that inhibiting P2X7 could ultimately enhance cognitive function in AD by rescuing synaptic plasticity and promoting neuronal survival. The effect of knockout or inhibition of P2X7 was initially assessed on neuronal impairment in AD mouse models. Firstly, ex vivo electrophysiological experiments conducted on acute hippocampal slice preparations have demonstrated that the absence of P2X7 rescues deficits in long-term synaptic plasticity in APPPS1 mice and long-term synaptic plasticity and depression in THY-Tau22 mice [20,23]. APPPS1 mice showed synaptic damages as assessed by PSD95 immunoreactivity in the hippocampus, which was rescued in the absence of P2X7 [20]. In addition, P2X7 inhibition or knockout also rescued the loss of hippocampal neurons in P301S mice, an effect in part mediated by preventing impairments of the ubiquitin-proteasome system [24,46].

Consistent with these findings, several behavioral tests have shown that inhibiting P2X7 effectively reduces cognitive impairments observed across these AD models. Long-term spatial memory performance assessed through the Morris water maze test revealed that a lack of P2X7 rescues memory deficits in both amyloid APPPS1 and tauopathy THY-Tau22 mouse models [20,23]. In P301S mice, a genetic or pharmacological blockade of P2X7 using GSK1482160 restored cognitive deficits in Y-maze and novel object recognition tests [24,45], while increased levels of P2X7 exacerbated memory deficits [24]. Therefore, inhibiting P2X7 not only reduces pathological AD lesions but also improves synaptic plasticity in the hippocampus and rescues memory deficits.

### 3.6. P2X7 and the Inflammasome NLRP3

Both in vitro and in vivo experiments have provided evidence that the activation of the inflammasome NLRP3 contributes to the pathological inflammatory response mediated by Aβ peptides [47,48]. One critical component of this inflammatory response is the Aβ-induced secretion of IL-1β [49]. P2X7 has emerged as a major player in NLRP3 activation and has been shown to contribute to the release of IL-1β in a wide range of models of neurodegenerative diseases [8,50]. Specifically, in models of AD, P2X7 plays a key role in Aβ-induced IL-1β secretion [49,51]. Intra-hippocampal injection of Aβ peptides increases IL-1β levels in the brains of wild-type animals but not in P2X7 knockout mice [51]. Similarly, treatment of J20 mice with BBG resulted in a significant decrease in IL-1β levels [44]. Also, in P301S mice, both the pharmacological blockade and knockout of P2X7 reversed the increase in IL-1β levels observed in this model [24]. Altogether, these findings suggest that P2X7 may regulate IL-1β release in AD. Mechanistically, the Aβ-induced secretion of IL-1β is mediated by microglia, and, indeed, the microglial release of IL-1β is inhibited by the P2X7 antagonist oATP and P2X7 knockout [49,51].

However, inhibiting P2X7 does not ameliorate the pathological inflammatory response in all AD models. For example, the cerebral levels of IL-1β were similar between APPPS1 mice and P2X7-deficient APPPS1 mice [20]. Moreover, no differences were observed in the mRNA levels of each component of the NLRP3 complex (Asc, Nlrp3, Caspase1, and Il-1β) between THY-Tau22 mice expressing or lacking P2X7 [23]. The differences observed in the involvement of P2X7-dependent IL-1β release in these models can be attributed to the varying levels of inflammation, notably the IL-1β level specific to each model. It is possible that in more inflammatory models such as intra-hippocampal injection of Aβ, J20, and P301S mice, P2X7 would have a significant role in the release of IL-1β, contrary to less aggressive models like APPPS1 and THY-Tau22 mice (https://www.alzforum.org/research-models/alzheimers-disease, accessed on 10 June 2023). Taken together, these findings suggest that P2X7 can regulate the pathological response associated with IL-1β in AD, especially in more inflammatory states.

### 3.7. P2X7 and Chemokines

Chemokines, a family of chemotactic cytokines, also participate in the development of AD [52]. The chemokines CCL3, CCL4, and CCL5 have particular importance in AD pathogenesis. These chemokines are upregulated in the brains of both amyloid (APPSwe/PSEN1dE9 and APPPS1) and Tau (THY-Tau22) models compared to control mice [53,54] and in the brain of AD patients [55]. Moreover, intracerebroventricular injection of Aβ peptides in mice induces increased levels of CCL3 and CCR5, the shared receptor for CCL3, CCL4, and CCL5. These chemokines play an important role in the loss of cognitive function in AD. The loss of CCL3 or CCR5 rescues the cognitive impairments and synaptic dysfunctions and reduces the inflammatory response induced by Aβ peptides [56]. Intracerebroventricular injection of CCL3 in mice impairs long-term plasticity and spatial memory, which can be alleviated by the CCR5 antagonist maraviroc [57]. The administration of maraviroc also reduces Tau hyperphosphorylation in THY-Tau22 mice [23].

The production of these chemokines in AD is P2X7-dependent, predominantly due to P2X7 activation on glial cells. P2X7 deficiency primarily impacts the production of CCL3, CCL4, and CCL5 in the brain of 15-month-old APPPS1 mice [20] and the expression of CCL4 in the hippocampus of 8-month-old THY-Tau22 mice [23]. The stimulation of glial cells, rat astrocytes, and microglia, with the P2X7 antagonist benzoyl-benzoyl ATP (Bz-ATP), induces CCL3 release that was reversed by P2X7 antagonists [58,59]. Through the use of P2X7-deficient mice, we demonstrated the specific involvement of P2X7 in the production of CCL3 in astrocytes and microglial cells and CCL4 in microglia in response to P2X7 agonists ATP and Bz-ATP [20,23]. Interestingly, we showed that Aβ peptides also induced CCL3 release but not from P2X7-deficient glial cells [20].

The P2X7-dependent production of chemokines could directly influence cognitive functions, but also, chemokines could contribute to cognitive impairments in AD through the recruitment of pathogenic T-cells. CCL3 is overexpressed in peripheral T-cells of AD patients and may facilitate T lymphocyte infiltration into the brain [60]. Pathological infiltration of CD8^+^ T lymphocytes in THY-Tau22 mice correlates with cognitive impairments, which can be improved by depleting these lymphocytes [54]. The improvement in cognitive impairment observed in P2X7-deficient APPPS1 mice could be attributed to the lower level of CCL3 production associated with a reduction in CD8+ T cells in the brain [20]. These results shed light on a new role of P2X7 in AD pathology through the production of chemokines.

### 3.8. P2X7 and Microglia

Although the increase in P2X7 levels in AD is well established, the cell types expressing P2X7 in the central nervous system have remained a subject of intense discussion for many years [61,62], primarily due to the lack of high selectivity of anti-P2X7 antibodies. Also, the amount of P2X7 can vary depending on the pathophysiological context, which adds complexity to the interpretation of the different studies. Different approaches have been developed to address this question, such as P2X7 transgenic reporter mice [21,63,64] and P2X7-specific nanobodies [64]. Currently, there is not much evidence for the role of P2X7 expression by neurons in AD, but more investigation is necessary to conclude more definitively the importance of neuronal P2X7 [61,62]. Using RNAscope ISH, we demonstrated an increase in P2X7 mRNA in microglia and astrocytes in a mouse model of AD [20]. Moreover, the P2X7 protein was detected in microglia and astrocytes in the cortex of patients with AD or frontotemporal lobar degeneration [20,23]. In general, microglia exhibit the highest levels of P2X7 in the brain [65,66]. Thus, the observed increase in P2X7 in glial cells suggests their potential contribution to the pathogenesis of AD.

**Figure 1:**
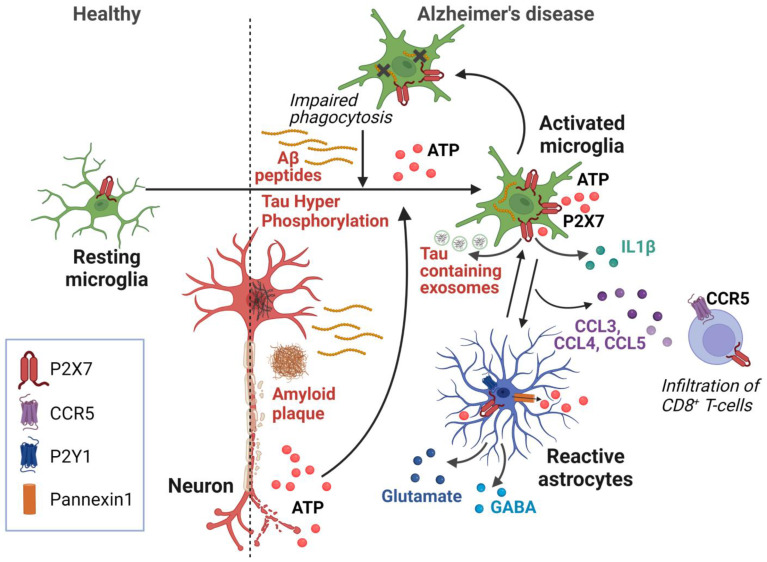
Schematic representation of P2X7-dependent mechanisms involved in Alzheimer’s disease. In this model, increased levels of Aβ peptide induce ATP release from microglia and astrocytes, as well as from damaged neurons. This ATP release is amplified by astrocytes via purinergic receptors and ATP release via Pannexin 1. P2X7 on microglia and astrocytes senses high ATP levels and initiates neurodegenerative processes via the production of IL-1β and CCL3, CCL4, and CCL5 chemokines, leading to impaired neuronal function and the recruitment of pathogenic CD8^+^ T lymphocytes. Activation of P2X7 also induces the secretion of Tau-containing exosomes from microglia, contributing to Tau pathology. The presence of high amount of ATP inhibits P2X7-mediated phagocytosis, further increasing the accumulation of Aβ peptides. Created with BioRender.com.

Several of the critical pathogenic functions of P2X7 could be mediated by P2X7 specifically expressed on microglia, including the release of IL-1β and chemokines and the secretion of Tau-containing exosomes (see previous paragraphs) (Figure 1 and Table 2) [20,23,24,44,45]. Thus, inhibiting microglial P2X7 could be beneficial for many facets of AD. The importance of microglial P2X7 is further underscored by the alternate role P2X7 can play in phagocytosis [16]. While the pro-inflammatory effects of P2X7 are predominantly mediated by the open state of the receptor, the scavenger activity of P2X7 is likely attributed to its closed state. Indeed, Gu et al. showed that P2X7 acts as a scavenger receptor in the absence of its ligand ATP [67]. Following ATP binding, the conformational change in P2X7 may induce the dissociation of the non-muscle myosin heavy chain from the P2X7 complex, which is needed for phagocytosis [16,68]. Furthermore, the inhibition of P2X7 in the presence of its ligand appears to reverse the P2X7 activation state and restore its phagocytic function [67]. Interestingly, the inhibition of P2X7 in a primary mouse microglial cell culture using siRNA or the antagonist BBG enhanced the phagocytosis of Aβ peptides in the presence of Bz-ATP [69]. In addition, in P301S mice, the inhibition of P2X7 with the antagonist GSK1482160A was found to increase the phagocytic capacity of microglia [24].

Under pathological conditions, in the presence of a high extracellular ATP concentration, the P2X7 function shifts from phagocytosis to the release of inflammatory mediators. In AD, we hypothesize that, partly due to aging, microglia become overwhelmed and are unable to be properly activated for the phagocytosis of extracellular aggregates. Instead, they release pro-inflammatory cytokines and chemokines that contribute to pathological processes, forming a vicious cycle [52,53]. Therefore, inhibiting P2X7 could regulate the polarization of microglia, shifting them from a pro-inflammatory state characterized by the production of cytokines and chemokines towards a state that promotes the phagocytosis of aggregates of Aβ peptides and abnormal extracellular hyperphosphorylated Tau proteins (Figure 1).

### 3.9. P2X7 and Astrocytes

In AD, astrocytes undergo changes that reduce their support for synapses and exhibit impairments in the endolysosomal pathway responsible for protein clearance [70]. P2X7 inhibition did not result in any observed changes in the morphology of astrocytes in AD mouse models [20,23,24,44], but further phenotyping of the heterogeneous activated astrocyte population is required to investigate this in depth. Still, several lines of evidence suggest that P2X7 expressed by astrocytes could potentially be involved in pathological processes during AD [70]. First, the P2X7 protein was detected in astrocytes within the brains of AD patients, particularly in the vicinity of amyloid plaques, and also in the brains of patients with frontotemporal lobar degeneration and Pick’s disease [20,23,24]. Further, when astrocytes are stimulated with ATP or Bz-ATP, the release of CCL3 was only observed in cells of wild-type mice but not of P2X7-deficient mice [20]. As discussed above, the genetic deletion of P2X7 in mouse models of AD and tauopathy has shown positive effects on cognitive functions compared to wild-type mice. Upon stimulation of hippocampal brain slices with the agonist Bz-ATP, the release of both glutamate and/or GABA was observed [71], yet this effect was found to be associated with the presence of P2X7 specifically in astrocytes rather than neurons, indicating their potential involvement in the excitotoxic effects of glutamate and neuronal inhibition in AD [72,73] (Figure 1 and Table 2). Further research is needed to fully understand the potential role of P2X7 expressed by astrocytes in the context of AD.

### 3.10. P2X7 as a Therapeutic Target

Inhibiting P2X7 is a promising avenue for the development of therapeutic interventions for AD [74]. As discussed above, P2X7 is upregulated during AD, and in many models, inhibiting or knocking out P2X7 ameliorates the P2X7-driven inflammation and rescues critical AD-related deficits. In the central nervous system, P2X7 is the most abundant on glial cells, especially microglia [65], and current evidence suggests these are the most important cells underlying pathological P2X7 functions, so it is assumed that the effects of P2X7 inhibitors will largely be mediated by inhibiting microglial P2X7 [66]. However, these effects need to be more thoroughly elucidated with a cell-specific inhibition of P2X7 and will undoubtedly be the subject of future studies.

Previously, although these therapies did not reach clinical availability, P2X7 antagonists have been investigated as a treatment for rheumatoid arthritis [75,76] and, more promisingly, Crohn’s disease [77], which found the P2X7 inhibitors to be safe and well tolerated. The development of P2X7-targeting therapies for AD can build upon these foundations. The low sensitivity of P2X7 makes it a particularly attractive pharmacological target because its lack of response in physiologically normal extracellular ATP levels suggests that inhibiting P2X7 should only have an effect when extracellular ATP levels spike to pathological levels [66]. Pharmacological antagonists are one potential tool for inhibiting P2X7 in disease. Initially, BBG was considered a potential therapy targeting P2X7, but it has largely been abandoned as a therapeutic option due to it also inhibiting voltage-gated sodium channels [78,79]. More recently, other more specific compounds with excellent blood–brain barrier (BBB) permeability have been developed [17,80,81]. However, they have not yet been the focus of clinical trials.

Another tool that could be used to inhibit pathological P2X7 is blocking antibodies, particularly nanobodies. In fact, there are nanobodies currently under development and validation [82]. Although their potential use in AD remains unexplored, there are particularly promising pre-clinical effects in a mouse model of stroke, another neurological disorder in which increased extracellular ATP acts as a critical danger signal and where microglial P2X7 plays an important pathological role [83]. Unlike BBB-permeable inhibitors, one limitation of nanobodies is that the delivery method may be more invasive because nanobodies do not as readily pass the BBB. However, nanobody-encoding AAVs are a promising strategy to more stably express nanobodies in the brain, thus requiring fewer injections while more specifically targeting the brain [82].

## 4. Conclusions

The investigation of P2X7 in a pathological context remains a complex area of study, primarily due to its diverse functions, which depend not only on the expressing cells but also on the surrounding environmental conditions that can modulate its activation state. Despite this complexity, many studies in vivo, in vitro, and in patients all suggest that P2X7 activation plays a role in several pathological processes in AD, including the formation of lesions, cognitive impairments, and the accompanying inflammatory response. Notably, the pro-inflammatory function of P2X7, attributed largely to its levels in microglial cells, is well established and is a promising avenue for the development of therapeutics. However, the direct or indirect contribution of astrocytes and other cells to the P2X7-mediated processes needs to be further explored. Promisingly, numerous pharmacological and biological inhibitors, such as P2X7-blocking antibodies, are currently under development and validation [82,83]. As our ability to detect and diagnose AD at earlier stages improves, the incorporation of P2X7-targeting therapies has the potential to dramatically improve the treatment of AD.

## Figures and Tables

**Table 1:** Summary of key *P2X7* SNPs with relevance to AD.

SNP	rs Number	Amino Acid Change	Affected Region of P2X7 Protein	Change in Function	Associated AD Risk	Reference
1513A>C	rs3751143	Glu496Ala	Carboxyl-terminal tail	Loss of function	Decreased risk of AD	[32]
489C>T	rs208294	His155Tyr	Extracellular loop	Gain of function	Increased risk of AD	[33]
474G>A	rs28360447	Gly150Arg	Extracellular loop	Loss of function	Increased risk of AD	[30]

**Table 2:** Key roles of P2X7 in the immunopathology of AD.

Role	Cells Involved	References
Aβ phagocytosis	Microglia	[16,24,69]
Release of Tau containing exosomes	Microglia	[45]
Release of IL1β	Microglia	[24,44,49,51]
Release of chemokines	Microglia	[20,23,59]
Release of chemokines	Astrocyte	[20,23,58]
Release of glutamate and GABA	Astrocyte	[70,71,72]

## Data Availability

No new data were created or analyzed in this study. Data sharing is not applicable to this article.
